# Research and Implementation of Mobile Internet Management Optimization and Intelligent Information System Based on Smart Decision

**DOI:** 10.1155/2021/5144568

**Published:** 2021-12-09

**Authors:** Yanqing Han, Yuyan Lei, Zimin Bao, Qingyuan Zhou

**Affiliations:** ^1^Xi'an Jiaotong University City College, Xi'an, China; ^2^College of Economics and Management, Xi'an University of Posts & Telecommunications, Xi'an, China; ^3^School of Economics and Management, Changzhou Vocational Institute of Mechatronic Technology, Changzhou, China

## Abstract

The way by which artificial intelligence is implemented is similar to the thinking process of the human brain. People obtain information about external conditions through five senses, namely, vision, hearing, smell, taste, and touch, and, through the further processing of the brain, it forms meaningful decision-making elements. Then, through the process of analysis and reasoning, further decisions are made. In the information age, the application of intelligent management information systems in various fields has promoted the modernization and intelligence of social development. From the perspective of intelligent decision-making, this paper analyzes the requirements of intelligent information systems and designs an intelligent information system based on mobile Internet management optimization, including system management optimization, and proposes an environment-based layer, network transport layer, and the three-tier system architecture of the smart service application layer. Finally, this paper considers the problem of data fusion after system expansion. According to the existing fuzzy fusion algorithm, a weight-based fuzzy fusion algorithm is proposed. The simulation analysis shows that the algorithm can be effectively applied in intelligent information systems.

## 1. Introduction

Artificial intelligence (AI) is an important branch of computer science. It attempts to understand the essence of intelligence and produce a new intelligent machine that can respond in a similar way to human intelligence. The research in this field includes robot, language recognition, image recognition, natural language processing, and expert system. Artificial intelligence can simulate the information process of human consciousness and thinking. Artificial intelligence is not human intelligence, but it can think like people and may exceed human intelligence. It is also considered as one of the three cutting-edge technologies in the 21st century. In the past few years, some computer systems with artificial intelligence have been established to control spacecraft and underwater robots [1]. Through the program, people can make some thinking reasoning, so that they have certain advanced intelligences such as environmental adaptation, automatic learning, and automatic decision-making [2]. In addition to the application of artificial intelligence to sensory simulation, a more important application is to simulate the thinking and analysis process of the human brain, namely, game and logical reasoning, the application of information sensing, and processing. In games or simulation systems, the application of such artificial intelligence is extraordinarily rich. Some techniques, applied in the chess program, such as looking forward a few steps and breaking down complex problems into some easy subproblems, are developed. It evolved into the basic technology of artificial intelligence such as search and problem summarization. At present, the project technology is developing rapidly and amazingly [3].The next generation of artificial intelligence system will affect our life more widely. Artificial intelligence will make more critical and personalized decisions for human beings through interaction with the environment.

The research goal of “3S Smart Service System” [4] is to build a universal access system based on technologies such as Internet of Things, cloud computing, and big data, which can accept intelligent industry, intelligent agriculture, intelligent logistics, intelligent transportation, smart grid, intelligent environmental protection, intelligent security, intelligent medical treatment and smart home, etc. Different types of IoT specific applications [5] provide common solutions for different application scenarios of IoT technology and achieve unified management and control. Considering that the system will be built into an intelligent system with massive data, scientific control, intelligent services, etc., the system is particularly important for the storage, management, processing, and transmission of data. The secure storage, specification management, intelligent processing, and reliable transmission of service environment data will provide reliable guarantee for data volume and data accuracy for system computing and decision-making.

Aiming at the problems existing in intelligent products or systems, this paper intends to adopt a support system model based on intelligent decision-making to design an intelligent information system based on Internet management optimization. The research focuses on intelligent information systems to integrate different intelligent products into the same system and, through big data and cloud computing technology, provide users with truly intelligent, personalized, automated full-service, without the need to control the system through user behavior. Considering the massive data characteristics of the system expansion, according to the existing fuzzy fusion algorithm, a fuzzy fusion algorithm based on weight is designed to provide more accurate and reliable raw data for the decision module of the system.

The remains of this paper are organized into four sections.  Section 2 contains related works of our research field. In Section 3, we give the design of system model.  Section 4 covers on the experimental and results. In the last section, we draw the discussion and conclusion over our research.

## 2. Related Work

Decision support system (DSS) is a computer application system that assists decision-makers to make semistructured or unstructured decisions by human-computer interaction through data, model, and knowledge [7]. It is an advanced information management system produced by the development of management information system (MIS) to a higher level. It provides an environment for decision- makers to analyze problems, establish models, simulate decision-making processes and schemes, and call various information resources and analysis tools to help decision-makers improve decision-making level and quality. Because DSS requires decision-makers to participate, man-machine dialogue is used to manipulate data through models. What is supported is only the structured and clearly procedural part of the decision-making process. The core content of decision support system is human-computer interaction. In order to help decision-makers deal with semistructured and unstructured problems, identify objectives and environmental constraints, further clarify problems, generate decision schemes, and comprehensively evaluate decision schemes, the system should have stronger human-computer interaction ability and become an interactive system. As the decision-making environment becomes more complex, the limitations of DSS in decision support are becoming more prominent:

1. Decision support system uses a static model to operate the data through the model. DSS is a system that makes comprehensive use of a large amount of data, organically combines many models, and assists decision-makers at all levels to realize scientific decision-making through human-computer interaction. The role of the system in decision support is passive and cannot be based on the decision environment. Changes provide active support [8, 9].
2. Decision support system is modeled by the decision-makers and requires decision-making problems with procedural and clear computability [10, 11], which cannot support the unstructured problems that are common in decision-making.
3. Decision support system is not a general product, but a solution. Each enterprise should combine its own situation, clarify the management difficulties to be solved, and then analyze, design, develop, and implement the decision support system, so as to truly meet the needs of enterprise management decision-making. DSS is based on quantitative mathematical models and lacks corresponding support methods for qualitative, fuzzy, and uncertain problems in decision-making [12, 13].

Intelligent decision support system (IDSS) is a combination of artificial intelligence (AI) and DSS and applies expert system (ES) technology to enable DSS to more fully apply human knowledge, such as descriptive knowledge about decision-making problems and procedural knowledge in the decision-making process. Introducing AI technology into DSS is mainly through the combination of expert system and DSS and adding inference engine and rule base to DSS system. In the decision-making process, many knowledge amounts cannot be expressed by data or described by models, so there is no fixed way of expertise and historical experience. Researchers integrate AI technology into DSS, mainly through the combination of expert system and DSS, and add inference engine and rule base to DSS system. The rule base introduced by IDSS can store these knowledge amounts and provide important reference and basis for decision-making. IDSS can have many types of information bases: text base (TB), database (DB), arithmetic base (AB), model base (MB), and rule base (RB). The text library stores a large number of documents written in natural language. The database stores the field form of the key factors of things. Various models reflecting the essential relationship of information are stored in the model base. Rule base is the most refined form of knowledge. From the original unprocessed data to the processed information and then to the extracted knowledge, this evolution relationship of information is called evolution chain.

From the viewpoint of system level, IDSS can be technically divided into three levels:

1. The application layer is directly oriented to IDSS users. In this layer, decision-makers can determine the status and constraints of IDSS according to their own needs. Decision-makers conduct system dialogue and input relevant information through the user interface; DSS understands user requests and commands through information conversion, carries out system reasoning operation, and reflects the results to users through the output interface. The whole process is transparent to users.
2. Control and coordination layer, for the chief designer of IDSS: its basic unit is the control and coordination module of the system central library. The system engineer establishes the relationship between them through the standard interface of each library.
3. The basic structure layer is for professional programmers. Professional programmers implement each library through this layer, specifically defining the organizational structure and communication mode of each library, so as to complete the department management and external communication tasks of each library.

During the operation of IDSS, each module needs to call the upper bridge repeatedly, which is less efficient than using the low-level call directly. However, considering that IDSS only operates when senior managers make major decisions, its operation frequency is much lower than that of other information systems, and the environmental conditions of each operation are very different; it is completely worth sacrificing part of the operation efficiency in exchange for the efficiency of system maintenance [14–16].

With the development of computer and artificial intelligence technology, the research focus of IDSS has gradually shifted from expert IDSS to the research of IDSS model system, man-machine interface, knowledge processing unit, and distributed IDSS. Distributed IDSS introduces distributed artificial intelligence (DAI) technology on the basis of knowledge-based IDSS. Its main idea is to use agent and multiagent system technology in Da; that is, agents playing different roles are designed as intelligent agents of modules according to the main functional modules in IDSS [17]. Through the self-learning mechanism, it simulates the different steps of human intelligence to complete the decision-making task, so as to make scientific decision-making. Distributed IDSS mainly designs and establishes large-scale and complex intelligent decision support system supported by Internet [18].

Expert system tools and technologies can be integrated into decision support system to provide users with consulting environment to improve decision quality and complete functions that conventional decision support system cannot complete. However, expert system is different from decision support system. When using decision support system, users must have certain professional knowledge and skills for the problems they deal with; that is, users should know how to reason about the problems, what questions should be put forward, how to get the answer, and how to proceed to the next step. At this time, the decision support system can only assist users to make decisions. However, the expert system is different. It itself has the professional knowledge of experts in a certain field to solve problems. Users only need to put forward the facts and appearances of the problem to the expert system.

## 3. System Model Design

### 3.1. Intelligent Decision Support System

Since the emergence of the intelligent decision support system, due to the great potential of expert system technology in the field of management decision-making, researchers at home and abroad have conducted a lot of research, integrating tools and methods such as computer science and artificial intelligence with human decision-making processes [19]. Intelligent decision system supports structure and function [20]. Holsapple summarized the system's support ability and learning ability for decision-making process and divided IDSS into 4 categories [21]: no adaptive, passive support; no adaptive, which can provide active support; adaptive, passive support; adaptive, active support.

#### 3.1.1. Active Decision Support System

The traditional decision support system provides the corresponding data and model, and the user chooses the corresponding method and model. The decision process is completely controlled by the user. The system only completes the auxiliary computing function. Like many developing things, active decision support system (ADSS) is a developing concept. The intelligent decision support system has part of the domain expert knowledge. Human intelligence has the ability of actively supporting decision-making. ADSS can change its behavior with the user's decision-making process, which is an important milestone in the research of decision support system, which is generated by intelligent decision support system [7]. Active decision-making is an important feature of ADSS. By establishing a human cognitive model, ADSS can provide decision-makers with different choices in different problem solving stages, thus forming different problem solving paths. ADSS provides decision-makers with different method choices at different stages of decision-making problem solving by establishing human cognitive model. ADSS is based on human prior knowledge, but its premise is that the system runs in a static decision-making environment. Therefore, ADSS still has the limitation of poor adaptability in practical application. However, the research on ADSS lays a foundation for the proposal of adaptive decision support.

#### 3.1.2. Adaptive Decision Support System

ADSS relies mainly on human prior knowledge. The operating environment of the system is static. The domain knowledge and reasoning knowledge required for decision-making are known in advance. In fact, the decision-making environment is usually changeable, and the problem solving process is closely related to the decision-making environment. In relation to this, the knowledge that humans possess and can use for reasoning is also limited. Therefore, the above ADSS knowledge structure and function have many limitations. Adaptive decision support system is an important step toward better support decision. Adaptability is based on the environmental changes of the system and improves the ability of the system of dealing with problems. In order to overcome the limitations of ADSS in knowledge structure, data mining, data warehousing, case-based reasoning, and other data-driven decision support methods and machine learning techniques are adopted. Algorithms such as ANN, GA, and ROUGH SET attempt to discover knowledge that is critical to decision-making from a large amount of historical data and past experience, so that the system has the ability of adjusting its behavior over time and decision process changes, thus generating adaptive decision-making support system (ADSS) [22].

The researchers try to find the knowledge related to decision-making problems from a large number of historical data and past experience by using machine learning and case-based reasoning, so as to make the system have the ability of adjusting its behavior with the change of time and decision-making process. On this basis, people have carried out a lot of research on ADSS, including system structure adaptation, domain knowledge adaptation, and user interface adaptation. Adaptability and self-learning ability have become a main symbol of intelligent decision support system. ADSS has logic-based reasoning in addition to traditional process calculations and other forms of reasoning and also uses inductive reasoning to implement dynamic knowledge systems. Inductive reasoning is a kind of nonmonotonic reasoning, which can be limited or incomplete knowledge. The state introduces a complete state of knowledge. Through inductive learning, ADSS has a certain ability of innovating and can use inductive assertions as knowledge. When new contradictions are contradictory, the knowledge obtained by inductive reasoning can be overthrown, thus maintaining the consistency of knowledge [23].

#### 3.1.3. Decision Expert System

The decision expert system is a decision support system established by using expert technology. The decision expert system uses deductive reasoning and uses existing knowledge to derive conclusions. The correctness of the derivation process can be guaranteed. The problem of this system is that a complete axiom system is needed for the basis of reasoning. In fact, in the environment of uncertain, abrupt, and fuzzy information, this condition is difficult to achieve, so it is only suitable for the application of well-defined decision-making tasks. In recent years, the progress of artificial intelligence and expert system technology seems to break through the limitations of traditional decision-making expert systems; the application of nonmonotonic reasoning and qualitative reasoning technology is broadening the application scope of expert systems and has made some progress in combination with human intelligence [24].

#### 3.1.4. Holistic Decision Support System

The integrated decision support system is based on the adaptive decision support system and the decision expert system [7]. In the process of human expert decision-making, it faces incomplete, uncertain, and even conflicting knowledge, and human thinking often has nonprocesses. Sexuality generally leads to decision-making through the synthesis of various knowledge and processes. The integrated decision support system (HDSS) emerges to mimic human advanced intelligence and can make full use of human in process analysis, logical reasoning, and cognition and learning. And it has the advantages of knowledge innovation, so that the system's auxiliary decision-making ability transcends the stage of fact, reasoning, and learning and can support the decision-making problem of ill-structured structure [25].

The application of Internet technology in the field of decision support makes the decision environment have new characteristics; that is, the data in decision analysis are no longer concentrated in one physical location, but scattered in different departments or regions. The above discussion about the types of decision support systems is based on the main characteristics of the system, from the perspective of the development of system intelligence. Several types are mutually contained and complementary. More advanced models reflect the evolution of system intelligence [26, 27], Excluding other types of features. In the big data environment, distributed decision support system will get more and more attention. In fact, the research of intelligent decision support systems is showing a trend of integration [7]. Existing systems are generally hybrid systems formed by a combination of methods, with several models characteristics. Uncertainty is the key problem in the current research of artificial intelligence technology, and it is also the core problem throughout the whole process of big data intelligent decision-making.  Table 1 compares several IDSSs from the aspects of system learning ability, intelligent behavior, and decision-making methods.

### 3.2. Intelligent Information System Requirements Analysis

Since the beginning of the 21st century, the wave of globalization has driven the rapid development of the social economy, and the quality of human life has been continuously improved. Together with the rapid development of information technology, human beings have begun to pursue the information technology and intelligence of the living environment. Such powerful human subjective needs have spawned the birth of intelligent information systems. Although the intelligent information products have been developed over a long period of time, this paper has found that the existing smart products or systems still have the following shortcomings after investigating the existing products or systems in the market:

1. Manufacturer's products are singularly mass-produced.
2. The existing smart device manufacturers only carry out mass production of a single product and do not systematically carry out product design and mass production and do not form a unified industry standard or access standard resulting in a mixed market of products and systems. There is poor compatibility between the two; new products are difficult to access the system.
3. The system control layer does not achieve true intelligence.
4. In response to the above problems (1), many manufacturers have proposed their own intelligent system solutions, integrating different types of products into the system and providing decision control functions, realizing remote control, security alarm, and other functions. However, the decision-making control layer of the system is not intelligent, but the user behavior is used for control or decision-making; that is, the user needs to manually adjust it.
5. The pressure on data processing of system servers in the Internet of Things has increased dramatically.
6. Most of the existing intelligent products use the intelligent gateway for data forwarding, and all the original data is sent to the remote server for analysis, storage, processing, etc. After the user scale is expanded, all the user's original data is processed by the remote server. The pressure on servers is getting bigger and bigger, and the cost of expansion is getting higher and higher.

In view of the shortcomings of the above existing intelligent products or systems, the intelligent system studied in this paper hopes to make improvements and finally build a gateway that can provide a unified device interface, realize decision control intelligence, and include local data storage and processing functions. The intelligent information system of the device provides users with systematic, automated, intelligent, and personalized services.

### 3.3. Hierarchical Structure Design of Intelligent Information System

The structure of the Internet of Things itself is complex and diverse. The current structure of an Internet of Things is divided into three levels: the perception layer, the network layer, and the application layer.

The bottom layer of the Internet of Things is the perception layer, which is the basis for realizing the comprehensive perception of the Internet of Things. RFID, sensors, two-dimensional code, etc., are mainly used to collect device information with sensors and use radio frequency identification technology to achieve transmission and recognition within a certain range. The main function is to identify objects and collect information through sensing devices.

The network layer is located above the sensing layer and is a network device and platform that serves the aggregation, transmission, and preliminary processing of IoT information. Through the existing three-network or next-generation network NGN, the huge amount of data collected from the sensor network is seamlessly transmitted over long distances; it is responsible for the secure transmission of the information collected by the sensor and the analysis of the collected information, processing and providing the results to the application layer.

The application layer is the top layer of the Internet of Things architecture. It mainly solves the problem of information processing and human-machine interface and provides the information services that people need through data processing and solutions. The application layer directly contacts the user and provides users with rich service functions. The user customizes the required service information, such as query information, monitoring information, and control information, on the application layer through the smart terminal.

The application of Internet of Things technology in the service environment mainly focuses on home appliance automation and intelligent security systems. Home appliance automation is installing sensors in traditional household electrical appliances (such as air conditioners, TVs, and refrigerators), making them intelligent, nodding them, and accessing the Internet, so that users can realize remote control of home appliances in the sky. The intelligent security system is equipped with sensor nodes for monitoring fire, gas concentration, etc., at home, so that the user gets the first time alarm feedback when there is a fire in the home and can timely handle the danger or escape in time to avoid personnel casualties.

**Figure 1:**
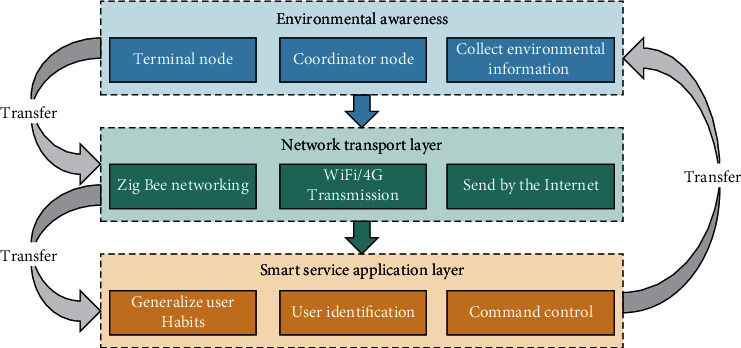
Intelligent information system hierarchy diagram.

The basic framework of an intelligent information system can be divided into three layers: the environment-awareness layer, the network transmission layer, and the smart service application layer as shown in Figure 1.

#### 3.3.1. Environment-Aware Layer

The environment-aware layer is the bottom layer of the system. If an analogy is made by one person, the environment-aware layer is like a human tactile nerve, which is composed of a large number of environmental information sensors. These sensors are like a neural node, distributed in every corner of the environment, collecting environmental information and sending the collected data to the upper layer of the intelligent information system through a certain short-range wireless transmission technology.

**Figure 2:**
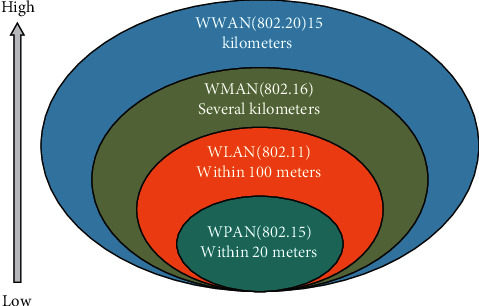
Correspondence between WPAN and other wireless networks.

From the perspective of network technology, the sensor of the environment-aware layer constructs a WPAN network within a certain range. The so-called WPAN is a network proposed to solve the “last few meters of wireless communication connection”. Generally, this refers to short-range wireless networks with coverage within a radius of 10 m, especially for self-organizing networks that can be connected shortly between portable consumer appliances and communication devices. WPAN is a network that is parallel with wireless wide area network (WWAN), wireless metropolitan area network (WMAN), and wireless local area network (WLAN) but has a smaller coverage. The corresponding relationship is shown in Figure 2.

#### 3.3.2. Network Transport Layer

The network transport layer is the middle layer of the intelligent information system of the service environment, which assists network communication and data transmission between the entire system levels. It includes communication between environmental information collection sensors, communication between environmental information collection sensors and terminals, and communication between terminals and smart cloud platforms.

#### 3.3.3. Smart Service Application Layer

The intelligent service application layer is the uppermost layer of the system and is the concentrated expression of the intelligence of the information system oriented to the service environment. It mainly includes the following functions:

- Cloud data storage and management
- Inducting user habits and building user knowledge base
- Smart decision based on user habits
- Sending control commands to the lower layer of the system

If one person is used as an analogy, the intelligent service application layer is equivalent to the human brain, which can store memory, intelligently think, make optimal decisions, and control other parts of the human body through neural networks. The intelligent service layer learns knowledge through machine learning algorithms and makes decisions to control the underlying devices of the system.

### 3.4. Improvement of Data Fusion Algorithm

The limitations of the fuzzy fusion algorithm in the current environment are mainly reflected in the two aspects of time efficiency and energy consumption. The improvement of the algorithm will also start from these two aspects: first, it reduces the amount of data to be fused by setting a reasonable threshold to improve the fusion algorithm by setting weights for data that fails to pass the threshold.

In an intelligent information system, assuming that there are *n* sensor nodes, they can form 2*n* − 1 sensor groups. As the number of nodes *n* increases, the number of sensor groups increases exponentially. Each sensor group performs the calculation of the tolerance function and the fuzzy measure function, which will generate a huge amount of computation and take a lot of time. Correspondingly, if we can reduce the number of *n* participating in the operation by a certain method, the calculation amount and time consumption of the algorithm will decrease exponentially. Therefore, reducing the amount of data with fusion is an effective way to solve the above problems.

Assuming that the system enters a stable data transmission phase, if the monitoring index does not fluctuate significantly over a period of time, then this part of the data is relatively small for system decision-making. The method designed in this paper is to record the result of each fusion and set an appropriate threshold. The data to be transmitted is compared and decided to be retained or deleted. The results of the reservation are directly involved in the final fusion, and the data to be deleted is given a weight, giving it the opportunity to reengage. The main process of the algorithm is as follows:

Suppose that the output set of *n* sensors is

(1) $$\begin{array}{c} X=\left[x_{1},x_{2},\ldots ,x_{n}\right]. \end{array}$$

After performing a fusion, the result of the fusion is recorded as *C*_0_, and, in addition, the threshold is set as

(2) $$\begin{array}{c} Thr=\left[C_{0}-\mu ,C_{0}+\mu \right]. \end{array}$$

In the next fusion, the node output *x*_*i*_ is compared with *Thr*. If *x*_*i*_ ∈ *Thr*, the data is deleted and the change output is *x*_1_, *x*_2_,…, *x*_*m*_, *m* ≤ *n*. There are three key points here: the selection of thresholds, the processing of deleted data, and the improvement of weight-based algorithms.

#### 3.4.1. Threshold Selection

Choosing the appropriate threshold is especially important in the improvement of the algorithm. If the threshold is selected to be too large, it will not be able to mask invalid data, and the accuracy of the data will be greatly reduced. If the threshold is selected to be too small, it will cause the data to be deleted and screened out in the next comparison, which will result in the algorithm. The amount of calculation and time consumption are not much different from those before the threshold is taken, and the meaning of the improved algorithm is lost. According to the above formula (2), the selection of the threshold in this paper is based on the result of the previous fusion C0. Here, we consider the fusion of temperature data.

**Figure 3:**
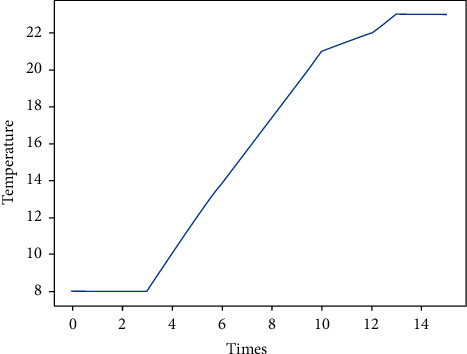
The room temperature rise curve.

Assume the following scenario: after the user enters the service environment, the room temperature is 8°C. The intelligent information system sets the air conditioner temperature to 25°C according to the user's habit. After the air conditioner is turned on, the room temperature rise curve is shown in Figure 3.

From the figure, we can see that, in the first few minutes, since the main engine has just started running after the air conditioner is started, the heating effect is not obvious and the temperature is almost changed. With the normal operation of the air conditioner main unit, the heating effect begins to appear and the temperature rises faster; later, as the room temperature has risen, the temperature rise requires more heat, so the temperature rise tends to be slow.

In this scenario, the fuzzy fusion of the temperature data is carried out. Obviously, the data in the first few minutes and the last few minutes shows a lot of redundancy, and some data can be eliminated by setting the threshold. After repeated experiments and measurements, this paper selects *μ*=0.5, which is the threshold of

(3) $$\begin{array}{c} Thr=\left[C_{0}-0.5,C_{0}+0.5\right]. \end{array}$$

#### 3.4.2. Delete Data Processing

In the above threshold elimination process, the data within the threshold range is directly deleted, but if the amount of data deleted is large, it will also cause distortion of the final fuzzy fusion result. In order to prevent this from happening, this paper introduces a processing mechanism for deleting data, which is to give the data a weight value to reflect its importance in the system.

The weight value mechanism of this paper is to assign a weight *W* to each data to be fused. The default value is *W* = 1. *W* represents the number of data amounts similar to the data and *W* = 1 means that the data can only represent itself.

**Figure 4:**
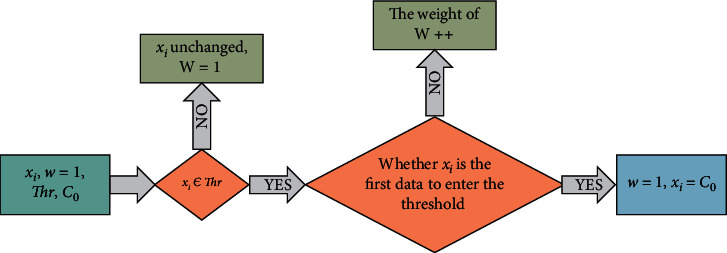
Data processing process that introduces weight values.

According to the above, assuming that the result of the last fuzzy fusion is *C*_0_, a weighted data to be merged *x*_*i*_ is compared with the threshold value (3), and if *x*_*i*_ ∈ *Thr*, whether it is the first data entering the threshold *Thr*, then, *C*_0_ is assigned to *x*_*i*_, and the weight *W* is still 1; the data that enters the threshold *Thr* again can be directly deleted, but each time a data enters the threshold the weight *W* of *C*_0_ is increased by 1, indicating that the number of data amounts similar to *C*_0_ is increased by one. If *x*_*i*_ ∉ *Thr*, the data is not adjusted, participating in the next fusion, and the weight *W* is still 1. This process can be represented in Figure 4.

#### 3.4.3. Weight-Based Algorithm Improvement

Since the weight of the data to be merged is introduced, the fuzzy measure function of the traditional fuzzy fusion algorithm cannot be directly used, and corresponding improvement is needed. The following describes the improvement of the fuzzy measure.

The fuzzy measure function reflects the reliability of the sensor group participating in the calculation. The range is between [0, 1]. The more the number of sensors participating in the fusion, the larger the fuzzy measure value and the higher the reliability. Otherwise, the number of participating sensors is less than the fuzzy measure value and thus the reliability is lower.

Assume, that in the home service environment, the number of sensors in the sensor group is *n*. When the number of participating sensors is *m* when a data is fused, the reliability can be expressed as *m*/*n*.

The result of the previous fusion is *C*_0_. After the next fusion, all data is compared with the threshold *T*, and the weight of *C*_0_ is *W* = *i*.

If *C*_0_ is not included in the sensor group data to be calculated, *m*/*n* is still used as the result of the fuzzy measure. If *C*_0_ is included in the sensor group data to be calculated, the fuzzy measure of the sensor group can be expressed by

(4) $$\begin{array}{c} \frac{\left(m+i-1\right)}{n}. \end{array}$$

The improved fuzzy measure guarantees the integrity of the data and makes the fusion result more accurate.

## 4. Designs and Analysis

### 4.1. System Architecture Design

**Figure 5:**
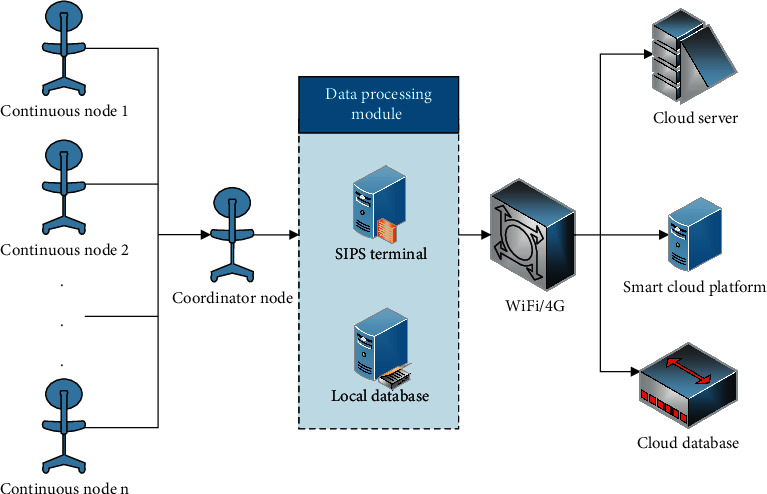
Intelligent information system architecture diagram.

This paper participates in the design of the presidential architecture of a service-oriented intelligent information system, as shown in Figure 5.

**Figure 6:**
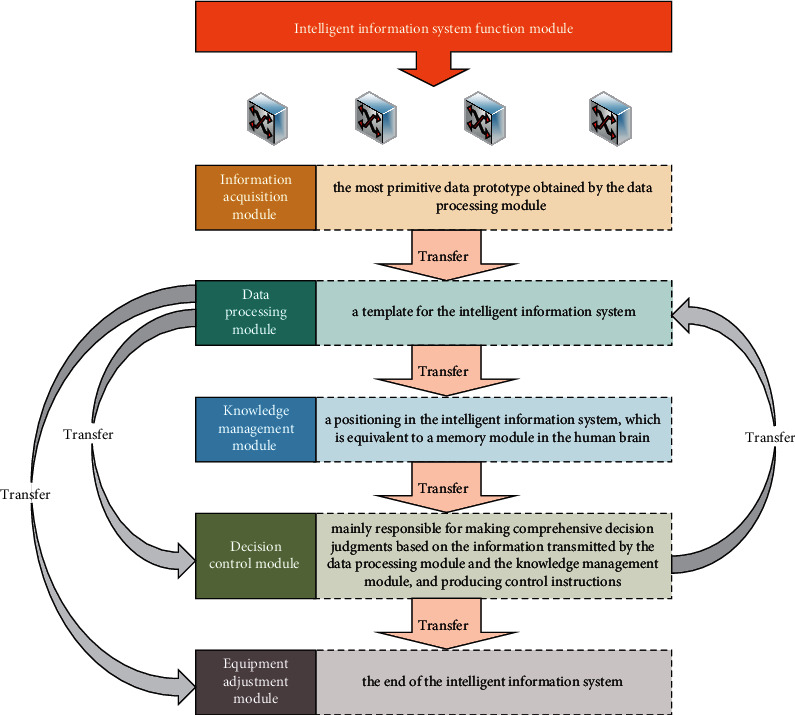
Intelligent information system function module diagram.

The intelligent information system is divided into functional modules, and the specific functions are designed. The functional modules include information collection template, data processing module, knowledge management module, decision control module, and device adjustment module. Their relationship to each other is shown in Figure 6.

The data collected by the information acquisition module is the most primitive data prototype obtained by the data processing module. It is the nerve ending of the entire intelligent information system and collects the information of the surrounding environment as accurately as possible for system processing, calculation, and decision-making.

The data processing module is a template for the intelligent information system. First, data is collected as input, then stored locally, and then preprocessed to provide a more accurate and reliable data prototype for the knowledge management module and decision control module.

The knowledge management module is a positioning in the intelligent information system, which is equivalent to a memory module in the human brain. Its input mainly comes from the data prototype provided by the data processing module, and the output is the user habit obtained by massive data accumulation and data mining. These data are stored in the database together with the data prototype and, on the other hand, as decision control. The data also can be input to the module for reference by the decision control module.

The decision control module is mainly responsible for making comprehensive decision judgments based on the information transmitted by the data processing module and the knowledge management module and producing control instructions.

The device adjustment template is one of the ends of the intelligent information system, receives the control command of the decision control module as an input, and performs input information to control the device parameters.

### 4.2. Experimental Analysis of Improved Data Fusion Algorithm

In this section, by setting the specific application scenarios, the data is simulated by MATLAB, and the traditional fuzzy fusion algorithm and the improved algorithm are compared and analyzed.

#### 4.2.1. Comparison of the Number of Participating Nodes

**Figure 7:**
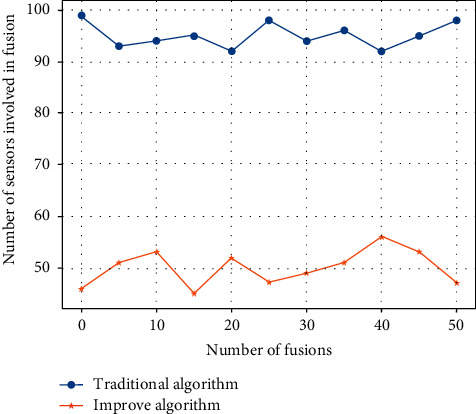
Comparison of the number of participating nodes.

First of all, this paper separately compares the number of sensors participating in each fusion of the two algorithms and obtains the results of Figure 7 as follows.

In Figure 7, the blue dot curve represents the traditional data fusion algorithm, and the orange dot curve represents the improved fuzzy fusion algorithm. As can be seen from the figure, in the traditional algorithm, the number of nodes participating in the fusion has been fluctuating around 90∼100; this means that, in every fusion process, almost all the data collected by the nodes will participate, which will cause huge calculations and consume system resources and time. The improved fusion algorithm has dropped significantly by half since the second fusion and then basically maintains a stable fluctuation around 50. This shows that, from the second time, the number of nodes participating in the fusion is significantly reduced. That is, setting a reasonable threshold makes the number of nodes participating in the fusion greatly reduced. From the previous analysis, we already know that when there are *n* nodes participating in the fusion, 2*n* − 1 kinds of node combinations will be generated. When *n* is reduced, the number of node combinations will decrease exponentially, which will greatly reduce the calculation amount of the system and improve the timeliness of data processing. Therefore, in the improved fuzzy fusion algorithm, it is feasible and effective to reduce the amount of data to be fused by setting a reasonable threshold.

#### 4.2.2. Comparison of Fusion Results

**Figure 8:**
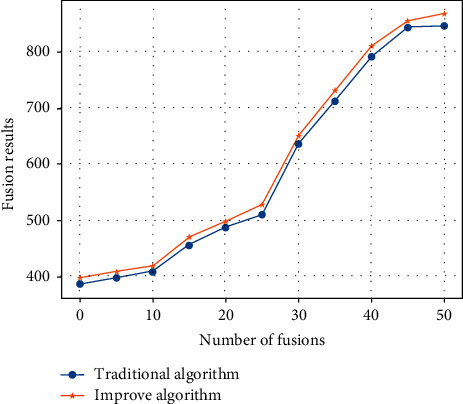
Comparison of fusion results.

At the same time, the fusion results of the traditional fuzzy fusion algorithm and the improved weight-based fuzzy fusion algorithm are compared. The comparison results are shown in Figure 8.

In Figure 8, the blue dot curve represents the temperature fusion result of 50 fusions by the traditional fuzzy fusion algorithm, and the orange dot curve represents the temperature fusion result of the improved weight-based fuzzy fusion algorithm. It can be seen from the figure that the curve of the improved algorithm is more consistent with the curve of the improved algorithm and the error is smaller. This shows that the improved algorithm can still accurately integrate the data and the improved algorithm is accurate and reliable.

## 5. Conclusions

Since the introduction of the concept of intelligent decision-making information system, after years of research and development, it has gradually entered the practical stage of the market. With the rapid development of technologies such as Internet of Things, big data, and cloud computing, its research has gradually focused on the wisdom of the system level. In view of the shortcomings of existing intelligent products or systems, this paper designs a data processing module of intelligent information system, considers the problem of data fusion after system expansion, and improves the data fusion algorithm. According to the analysis of requirements, the intelligent information system is designed, and the three-layer architecture of the system based on intelligent decision-making is proposed, including the sensing layer, the network transmission layer, and the intelligent service application layer. The module construction is decomposed to explain how the modules interact with each other to cooperate in work. Considering the massive data problem after the expansion of intelligent information system, the data fusion algorithm is compared and improved according to the traditional fuzzy fusion algorithm. A weight-based fuzzy fusion algorithm is proposed to make it more adaptable to massive data and improve the accuracy and reliability of system data. Through the simulation diagram, the improved algorithm and the traditional algorithm are compared and analyzed, and the effectiveness of the weight-based fuzzy fusion algorithm is verified.

## Figures and Tables

**Table 1:** Comparison of IDSS models.

System	Knowledge base type	Completeness of knowledge	Knowledge innovation ability	Decision process understanding	Main reasoning	Main decision-making tool
Active decision support system	Static	Completeness without conflict	No	Have	Adopt a predefined process	Data, cognitive model
Decision expert system static	Static	Completeness without conflict	No	Maybe do not have	Deductive reasoning	Knowledge base
Adaptive decision support system	Dynamic	Allowing incompleteness and conflict	Yes	Generally have	Deductive and inductive reasoning	Data, intelligent decision model, and knowledge base
Integrated decision support system	Dynamic	Allowing incompleteness and conflict	Yes	Have	Deductive, inductive, and case-based reasoning	Cognitive model, intelligent computing method, machine learning, and knowledge base

## Data Availability

The authors will make data available on request through a data access committee.
